# A Bibliometric Study on Trends in Proton Exchange Membrane Fuel Cell Research during 1990–2022

**DOI:** 10.3390/membranes12121217

**Published:** 2022-12-01

**Authors:** Zhijun Deng, Baozhu Li, Jinqiu Gong, Chen Zhao

**Affiliations:** 1 Research Institute of New Energy Vehicle Technology, Shenzhen Polytechnic, Shenzhen 518055, China; 2 Internet of Things & Smart City Innovation Platform, Zhuhai Fudan Innovation Research Institute, Zhuhai 518057, China

**Keywords:** bibliometric analysis, proton exchange membrane fuel cell, VOSviewer, collaboration network, visualization mapping

## Abstract

Proton exchange membrane fuel cell (PEMFC) with high density and safe reliability has been extensively studied in the world. With the circumstance of extensive PEMFC research, in this study we carried out a bibliometric analysis to understand the technological development. The information of 17,769 related publications from 1990 to 2022 was retrieved from the Web of Science Core Collection for bibliometric analysis based on the VOSviewer tool. The results show that the International Journal of Hydrogen Energy dominates among all of the source journals. The closest collaboration is between China and the USA, and publications from both of those account for 53.9% of the total. In terms of institutions, the Chinese Academy of Sciences has prolific publications, in which representative groups, such as Shao Zhigang’s, have achieved many outputs in this field. The theme of PEMFC research can be divided into three aspects: “materials”, “design” and “mechanisms”. This study demonstrated overall mapping knowledge domain and systematic analysis, and contributed to making a guide for researchers on the progress and trends of PEMFC.

## 1. Introduction

From the perspective of energy saving and ecological environment protection, fuel cells are the most promising power generation technology and have been attracting worldwide attention [1,2,3]. According to the types of electrolytes, fuel cells can be divided into alkaline fuel cells, proton exchange membrane fuel cells, nitric acid fuel cells, carbonic acid fuel cells, solid oxide fuel cells and so on [4,5]. Among them, the proton exchange membrane fuel cell (PEMFC) has advantages in high energy efficiency and density, small volume and weight, short cold start time, and safe and reliable operation, all of which have become the mainstream development directions of current fuel cell technology [6,7]. Recently, PEMFCs have been widely used in the automobile vehicle, ship, aerospace, energy generation, household power supply and other industries [8,9,10,11].

A single PEMFC is mainly composed of one proton exchange membrane (PEM), two electrodes, two bipolar plates, two gas diffusion layers and sealing gaskets [12]; there are numerous studies about these parts. First of all, new functional materials preparation has been a major trend. The development of mesoporous PEM [13,14,15,16,17] and new materials progress on bipolar plates [18], electrodes [19] and gas diffusion layers [20] were reviewed. On the basis of materials research, the analysis methods of diagnose technologies and module simulation were proposed. For instance, X. Zhang et al. introduced the lasted common electrochemical methods and physical/chemical methods, including cyclic voltammetry (CV), electrochemical impedance spectroscopy (EIS), pressure drop measurement, gas chromatography, neutron imaging, gas chromatography, etc. [21]. T. Jahnke et al. reviewed the modeling of PEMFC performance from the atomic scale to the system level, and the degradation mechanism [22]. This demonstrates the mature stage of the numerical model in the development of PEMFC research. In other respects, the reaction and degradation mechanism also has been elaborated by researchers, and improvement strategies, such as gas purge and water management, were provided [23,24,25].

Bibliometric studies are increasingly used for research analysis, which contributes to evaluating unlimited amounts of publications from institutions or countries and achieve their impact, and provide a knowledge structure for researchers to acquire information and conduct scientific communication [26,27,28]. For the research topic of PEMFC, some scholars have attempted to investigate its development with the bibliometric method. Compared with traditional reviews, it is more convenient to carry out a more comprehensive scientific review work from authors, institutions and other aspects through the bibliometric method. Bibliometric study covers a wider range of contents, which can be used to identify high quality works and guide research directions to a certain extent, assisting researchers to make better judgements on the trends of PEMFC based on quantitative results. Solis et al. demonstrated the development of mass transport in a gas diffusion layer in PEMFC over the last ten years, based on bibliometric analysis and Methodi Ordinatio. [29]. Yonoff et al. carried out a bibliometric analysis of the trends of PEMFCs research from 2008–2018 [30]. Lijun Wang also conducted a literature review on the development process of prognostic and health management (PHM) in the PEMFC, and proposed the future application direction of PHM should be diversifying [31].

However, so far, bibliometric methods have only been used to analyze the research of PEMFCs published in recent years, which involves a short time span and a single research direction in PEMFC. Therefore, in this paper, we carried out a more comprehensive bibliometric analysis of PEMFC research from 1990 to the present. This study can help junior researchers quickly identify the topics and directions that they are most interested in when studying a large amount of literature. Furthermore, it may also provide clear guidance for researchers who want to understand PEMFC research hotspots and trends.

## 2. Data and Methods

### 2.1. Data Collection and Data Cleansing

**Figure 1:**
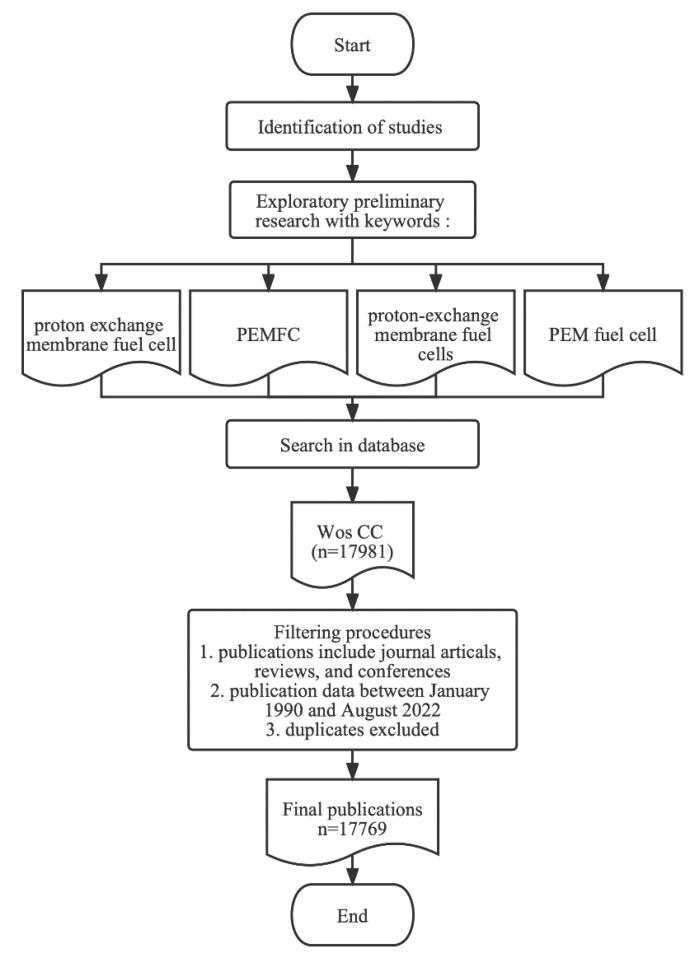
Flow chart of a systematic protocol.

Web of Science Core Collection (WoS CC) includes the world’s highest level of authoritative academic journals, monographs and conferences. It covers multiple disciplines and has a powerful index function for researchers to obtain global academic information. Compared with other databases, e.g., Scopus and Lens, WoS CC contains more comprehensive content and has an authoritative reputation for providing cite information, statistical analysis and evaluation, which have been more recognized by domestic researchers. Therefore, it is more suitable for this bibliometric analysis. Data for publications in this study were obtained by searching the WoS CC database for publications between January 1990 and August 2022. The edition is Science Citation Index Expanded (SCI-Expanded) from 1990 to the present. The search formula is TS = “proton exchange membrane fuel cell” OR TS = “PEMFC” OR TS = “proton-exchange membrane fuel cells” OR TS = “PEM fuel cell”, which produced a total of 17,769 publications with the classification “journal articles”, “reviews” and “conferences” after excluding duplicates. They were found and downloaded on 22 August 2022. The information from the metadata of the publications mainly consists of authors, titles, source journals, countries, institutions, publication years and keywords. Figure 1 shows the flow chart of the systematic protocol, indicating the whole path to obtain the data for this study.

Before the stage of keywords analysis, it is necessary to establish a thesaurus file for data cleansing. A thesaurus files is a text file to be imported into the VOSviewer when analyzing the co-occurrence of keywords. Some regular and indistinctive words for keywords analysis—such as “performance”, “fuel cell”, “proton exchange membrane”, “proton exchange membrane fuel cell”, etc.—are meaningless for the network map and need to be ignored during cleansing. Another circumstance is that some words are written in a different manner—e.g., “gas diffusion layer” and “gas-diffusion layer”, or “electrocatalyst” and “electrocatalysts”, or “dmfc” and “direct methanol fuel cell”—which may blur the picture and diminish the relevance of the visualized map. As a result, this type of data cleansing is supposed to unify singular and plural words, as well as replace synonyms.

### 2.2. Bibliometric Methods and Visualization Tools

Bibliometric analysis involves using mathematical and statistical methods to analyze and present the quantitative relations and rules of literature and literature work system, which can be further used to reveal the nature and the development of PEMFC [32]. In this article, the tool used for analysis is VOSviewer (version 1.6.18) (http://www.VOSviewer.com/, on 1 September 2022), which is developed by Nees Jan van Eck and Ludo Waltman [33,34]. VOSviewer software is used to analyze the structure and development of the science, which is applicable for a variety of scientific fields to display visualized collaboration network diagrams for bibliometric parameters [35,36,37]. In comparison with other bibliometric tools, VOSviewer has the advantage of excellent graphical presentation capability, which can be applied for large-scale data analysis and adapted to multiple database formats, such as Web of Science, Scopus and CNKI [38].

Based on bibliographic database files, VOSviewer software can build a co-authorship, keyword co-occurrence, citation, bibliographic coupling or co-citation map for units of authors, countries, organizations, keywords, sources and documents. Information about the number of items, clusters, links and link strength between items can be obtained from the map. Furthermore, according to the analysis results, VOSviewer provides three types of visualizations: network visualization, overlay visualization and density visualization. The parameters used for the map in this study are considered in Table 1, and the principles of VOSviewer in this study mainly contain co-authorship network, layout and clustering methods, as following [39].

(1) Co-authorship network

Based on full counting and fractional counting, in the “Create a map based on bibliographic data” functional module, VOSviewer can be applied to coupling analysis, co-occurrence analysis and co-authorship analysis. The equations related with the matrix multiplication for co-authorship analysis are as follows,

(1) $$u_{ij}=\overset{N}{\underset{k=1}{\sum }}a_{ik}a_{jk}$$

(2) $$u_{ij}^{*}=\sum_{k=1}^{N}\frac{a_{ik}a_{jk}}{n_{k}-1}$$

where the parameter of *i* and *j* is the document (the same with the following context), $u_{ij}$ represents the original cooperation intensity of *i* and *j* obtained by full counting method, and $u_{ij}^{*}$ represents the strength of the collaborative paper of *i* and *j* under fractional counting. In the author-paper matrix *A*, if *i* is the author of the paper *j*, then *a_ij_* = 1, otherwise *a_ij_* = 0.

(2) Layout method

The VOSviewer layout method is based on the distance-based maps method in order to determine the position of the element in two dimensions, that is, minimizing the weighted sum of squared Euclidean distances over all “pairs of elements”. The equations are demonstrated as follows,

(3) $$V\left(x_{1},\cdots ,x_{n}\right)=\underset{i<j}{\sum }S_{ij}\|x_{i}-x_{j}\|{}^{2}$$

(4) $$d_{ij}=\|x_{i}-x_{j}\|=\sqrt{\sum_{k=1}^{p}{\left(x_{ik}-x_{jk}\right)}^{2}}$$

(5) $$\frac{2}{n\left(n-1\right)}\sum_{i<j}\|x_{i}-x_{j}\|=1$$

where $x_{i}$ and $x_{j}$ refer to the spatial positions of *i* and *j*, respectively, and $d_{ij}$ represents the distance between two elements. To avoid overlapping positions among elements, the constraint that the average of the sum of distances of all elements is 1 needs to be set in Equation (5).

(3) Clustering method

Clustering divides the elements with high similarity into multiple groups. After completing the layout calculation, VOSviewer performs the clustering algorithm, which is specified in Equation (6).

(6) $$V\left(c_{1},\cdots ,c_{n}\right)=\frac{1}{2m}\underset{i<j}{\sum }\delta \left(c_{i},c_{j}\right)w_{ij}\left(c_{ij}-\gamma \frac{c_{i}c_{j}}{2m}\right)$$

In Equation (6), $w_{ij}=2m/c_{i}c_{j}$, $c_{i}$ is the cluster that *i* belongs to, the value of $\delta \left(c_{i},c_{j}\right)\;$is 1 (if $c_{i}=c_{j}$) or 0, and γ refers to the clustering resolution, whose value can be adjusted. The larger the value of γ, the more clusters that will be obtained.

The Scimago Graphica (https://graphica.app) tool combines diagrams’ interactions with a graphic processing function efficiently, and it was suitable for processing a single group of data [40,41]. Therefore, in this study, the data source Scimago Graphica is exported from VOSviewer. Then the Scimago Graphica tool is used to show the distribution of scientific output by country based on the country affiliation of the authors in a georeferenced form.

## 3. Results and Discussion

### 3.1. Journal Resource Analysis

**Figure 2:**
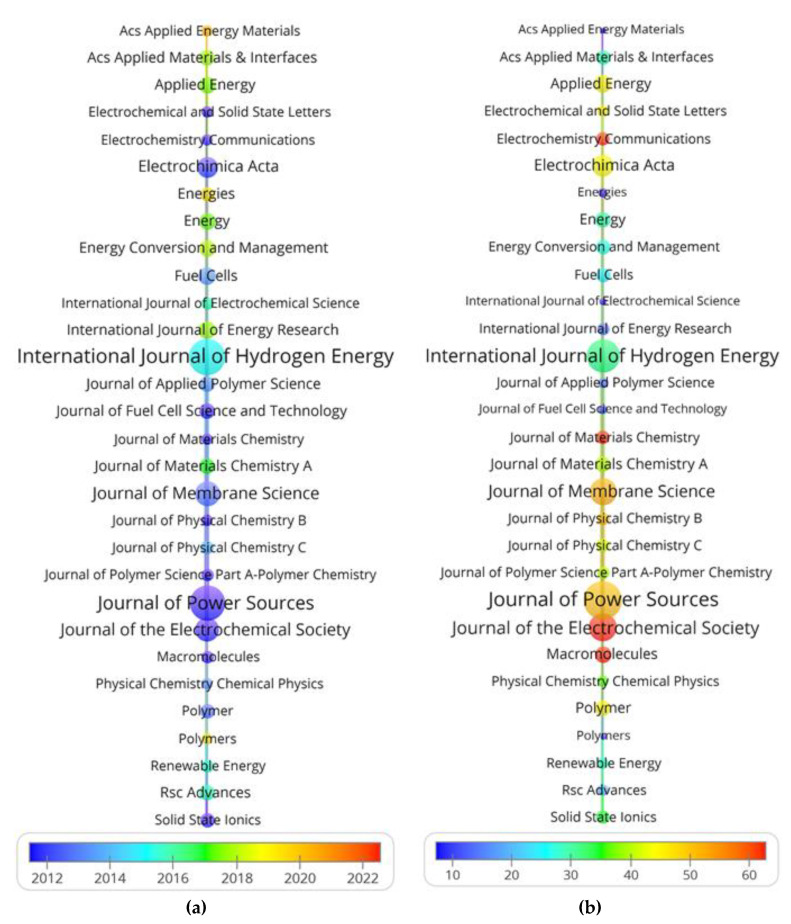
Source journal analysis of main journals (**a**) average publication year (**b**) average citations.

With respect to the theme of PEMFC, the documents, average publication year and citations of 30 journals, on the basis of statistics on journal source, can be visualized in Figure 2. The size of each circle refers to the number of documents of the journal and the color of each circle indicates the average publication year and citations, respectively. It seems that the average publication year of journals with a larger number of publications occurred during 2011~2015, and, accordingly, prolific journals receive more citations on average. In detail, the top 10 journals sorted by number of publications are listed in Table 2. The top three journals in term of number of publications are International Journal of Hydrogen Energy (2244), Journal of Power Sources (2078) and Journal of Membrane Science (770). The top two journals with respect to average number of citations are Journal of The Electrochemical Society (63.2) and Journal of Membrane Science (50.8). From the above factors, International Journal of Hydrogen Energy and Journal of Power Sources play the most significant role in disseminating and promoting research on PEMFC on the whole.

**Figure 3:**
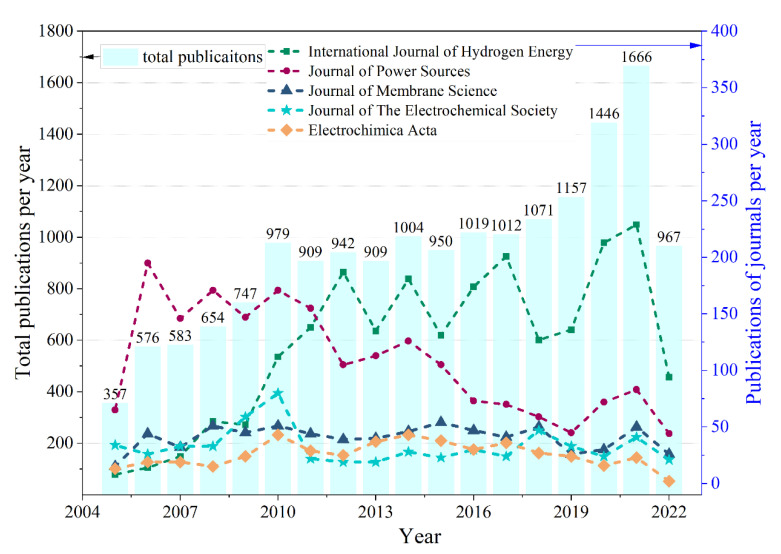
Number of total publications over time and amount comparison of annual publications of the top 5 journals.

In regards to journal source, the journals that have always dominated are International Journal of Hydrogen Energy and Journal of Power Sources, which both play an important role in the development of PEMFC. In a further step of the trend analysis for journals, the number of total publications over time and a comparison of the number of annual publications of the top 5 journals are shown in Figure 3. As of 2018, the number of publications has shown a steady upward trend over time, reaching a peak in 2021, which indicates that there has been a lot of research on PEMFC in recent years. It is obvious that the annual publications of International Journal of Hydrogen Energy show an increasing trend in the period 2005–2012. In addition, International Journal of Hydrogen Energy surpassed Journal of Power Sources in 2012 and has been in a leading position in terms of number of publications since then. In comparison, the publications Journal of Membrane Science, Journal of the Electrochemical Society and Electrochimica Acta present a stable trend and similar quantity.

### 3.2. Analysis of Global Authors and Collaboration Network

**Figure 4:**
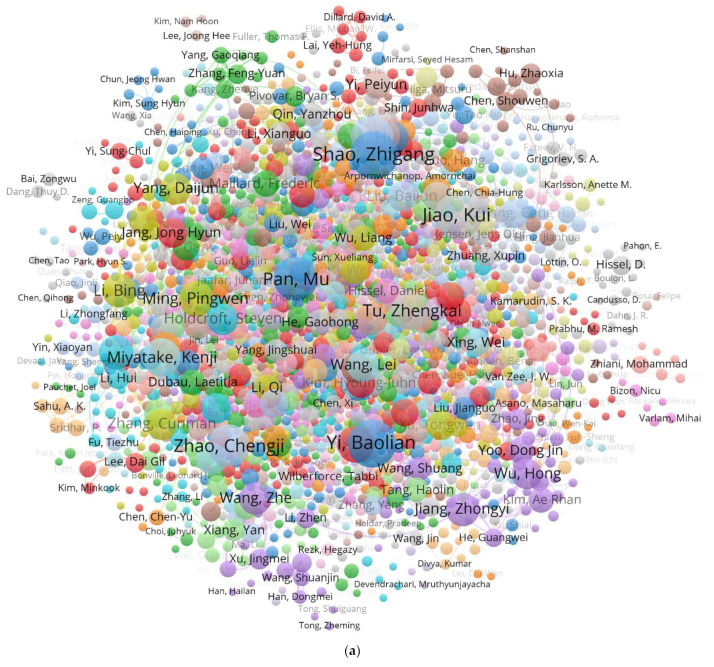

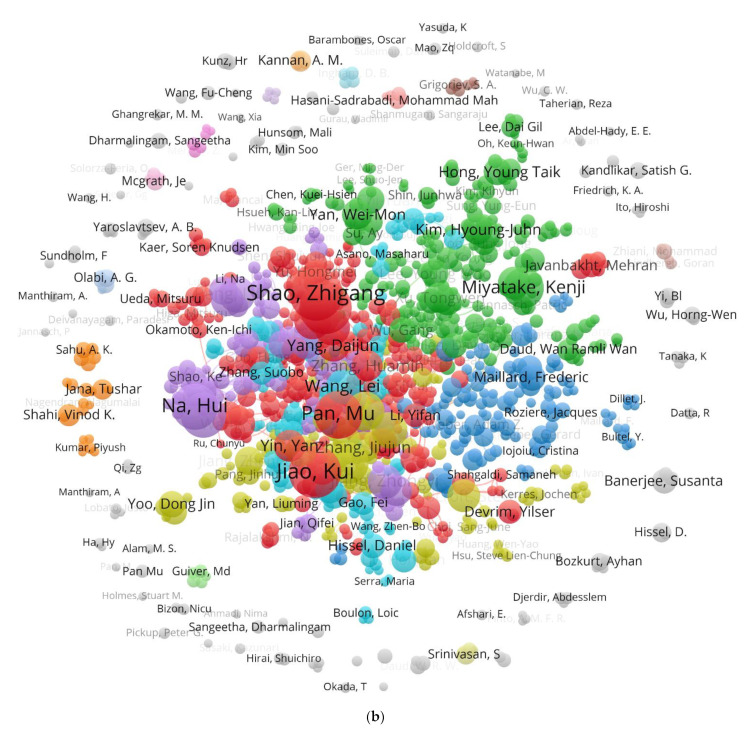

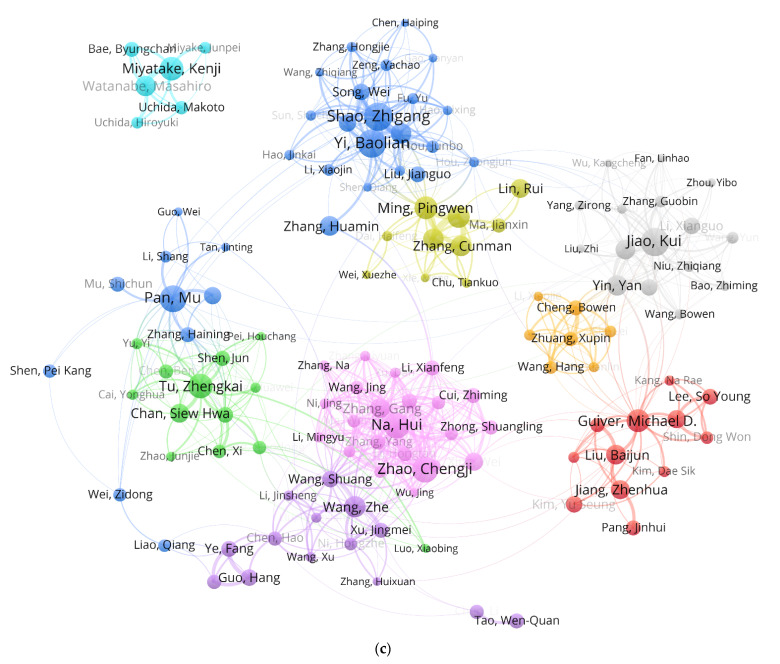
Collaboration network map of authors (**a**) the overall network (**b**) the network of main authors (**c**) the collaboration network of prolific authors.

In further analysis of the authors who have published documents in journals, the collaboration network of main authors ranked by the publication quantity via VOSviewer is shown in Figure 4. Figure 4a,b present the overall collaboration network of 2118 authors, as well as the collaboration network of 1076 main authors separately. As the network indicates, researchers from all over the world have been working on PEMFC with remarkable success. Moreover, there is a close relationship among authors of PEMFC research between those from China and those from other countries, based on both the global and the local network maps. Obviously, as shown in Figure 4a,b, there are some authors occupying conspicuous positions in the map. Therefore, it is necessary to analyze more information about those researchers.

The publication information of the top 10 authors mentioned above are listed in Table 3 by means of the VOSviewer tool. According to the publication ranking, only two researchers among the top 10 authors are not Chinese. As one of the top 3 representative Chinese authors, Zhigang Shao, whose group has the most publications in the major journals, has been engaged in developing new material on PEMFC with 116 publications [42]. Baolian Yi, with 113 publications, mainly conducts research on critical materials and the attenuation mechanism of PEMFC [43,44]; Yi also collaborates with Shao in the field of materials [45,46,47]. Finally, Kui Jiao, with 112 publications, mainly focuses on research on power machinery and engineering that provides solutions for thermophysical problems in PEMFC [48,49]. As for researchers who are not from China, Kenji Miyatake from Japan and Michael D. Guiver from the UK both work on the properties and structure of membrane materials, including proton-conducting membrane materials [50,51] and polymer-based membrane materials [52,53].

Based on the collaboration network of authors in Table 3, the map in Figure 4c demonstrates that the majority of authors are from China, and they maintain good relationships with each other. On the whole, the authors with the most representative clusters are the Shao and Yi research groups, both of which come from the Chinese Academy of Sciences, and their scientific interests mainly include new materials, critical materials and the attenuation mechanism of PEMFC. As for collaboration networks among authors who are not from China, Jiao has established relationships with Guiver, who has been invited to be an expert professor at the State Key Laboratory of Internal Combustion Engine Combustion at Tianjin University. Meanwhile, the network led by Miyatake from Yamanashi University is a self-contained cluster of co-authors from Japan and Korea. Owing to achievement in the field of proton conducting materials [54], Miyatake has been invited to China many times to participate in conferences on the subject of “energy and environment materials” for academic exchanges.

### 3.3. Quantitative Analysis of Countries and Institutions

**Figure 5:**
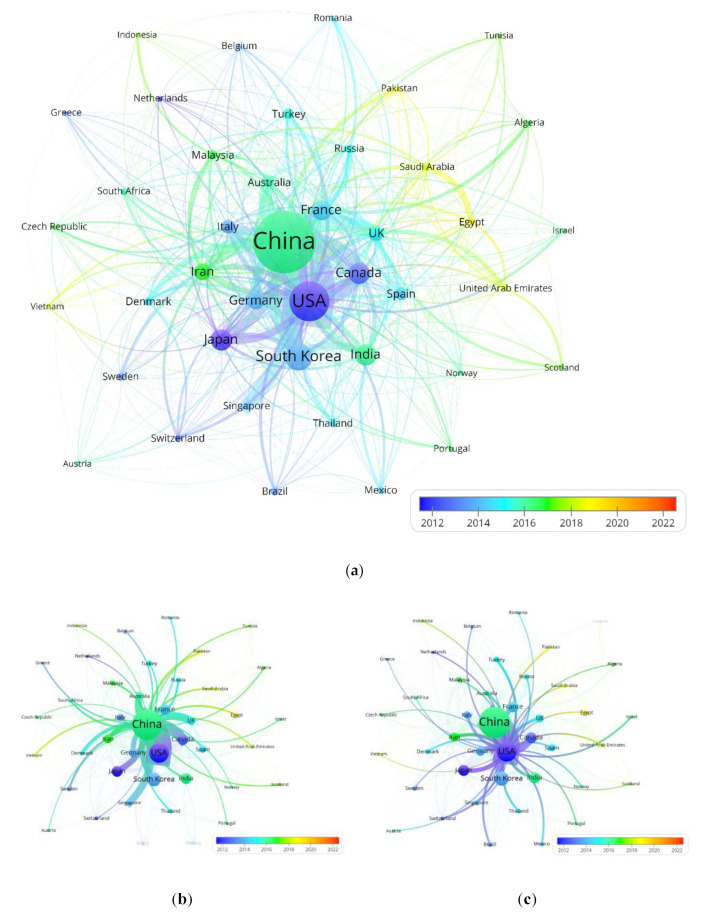
Collaboration network of main countries (**a**) overall collaboration network (**b**) collaboration network with China (**c**) collaboration network with USA.

Worldwide, countries have been contributing to research on PEMFC; consequently, the quantitative analysis of countries cannot be neglected for bibliometric study. Figure 5a shows the overall collaboration network of main countries in nearly a decade. It is apparent that there is extensive cooperation among countries, and China has the largest number of publications. According to the color of circles in the network map that refers to the average publication year of countries, it is evident that developed countries, such as the USA, Canada and Japan, have participated in the PEMFC research area earlier than China. In addition, due to the growing global energy crisis, some countries that have entered into this research field were attracted by developed countries in recent years, such as Pakistan, Egypt and Vietnam. Those countries have proposed a development plan for national hydrogen energy mission and provided substantial financial support to support the development of this technology. Figure 5b,c demonstrate the collaboration network of other countries with China and the USA, respectively. Viewed with respect to the progression of time, China established partnerships with other countries later than the USA, but it seems to have taken more advantage of publishing. From the aspect of the cooperation relationship, China has the closest cooperation with the USA, followed by Canada, the UK and Japan. In contrast, the major cooperation countries of the USA include China, South Korea, Canada and Germany.

**Figure 6:**
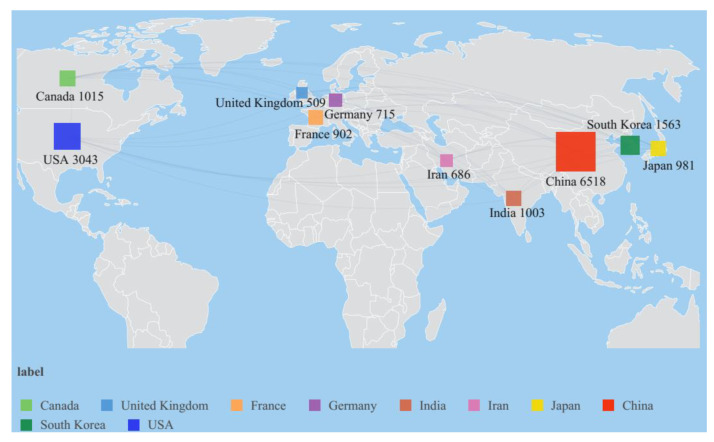
Map distribution of the top 10 countries.

Table 4 shows the number of publications, average citations (AC), average publication year (APY) and total link strength (TLS) of the top 10 countries. The above parameters in Table 4 reflect country participation in research on PEMFC during 1990–2022. For instance, the top 3 countries sorted by number of publication are China (6518, 36.7% of total publications), followed by USA (3043, 17.2%) and South Korea (1563, 8.8%). It is obvious that China and the USA have numerous outputs in the domain of PEMFC research all over the world. The average publication year is between 2012 and 2016, indicating that global PEMFC research is attractive and the output is high during this period. Moreover, the top 10 countries from Table 4 are from the Asia, North America and Europe regions. The combination with the geographical distribution of scientific output by the top 10 countries is displayed in Figure 6, and the number of publications by authors from China, South Korea, India, Japan and Iran in the top 10 countries accounts for 60.5% of the total. These are all Asian countries that are developing efficiently in the field of PEMFC.

**Figure 7:**
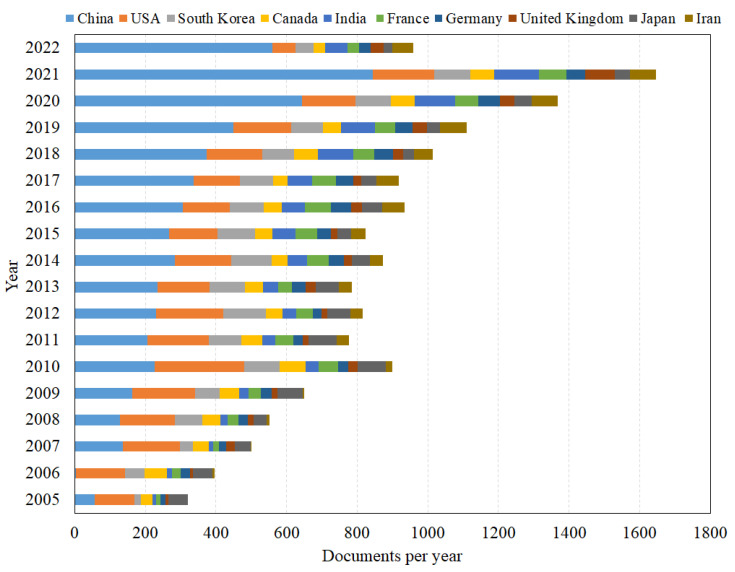
Trends of publications in top 10 countries.

The increasing trend of publications in the top 10 countries during the period of 2005–2022 is demonstrated in Figure 7. China and India, as developing countries, exhibit an increasing trend in publication quantity due to the development demands of domestic energy technology, whereas the USA and other developed countries maintain a relatively stable trend in comparison. China has become the most prolific country and has rated above the USA since 2012, and this phenomenon has been lasting until now. This turning point in time is probably related to the following two aspects. First, the China State Council issued a circular on “the Development Plan for Energy Conservation and New Energy Automobile Industry” (2012–2020), which emphasized the demand for research and development of energy-saving vehicle technology, including fuel cells. Second, Chinese researchers have an optimistic view and attitude on the development prospect of PEMFC, while continuing researching and applying new technologies.

**Figure 8:**
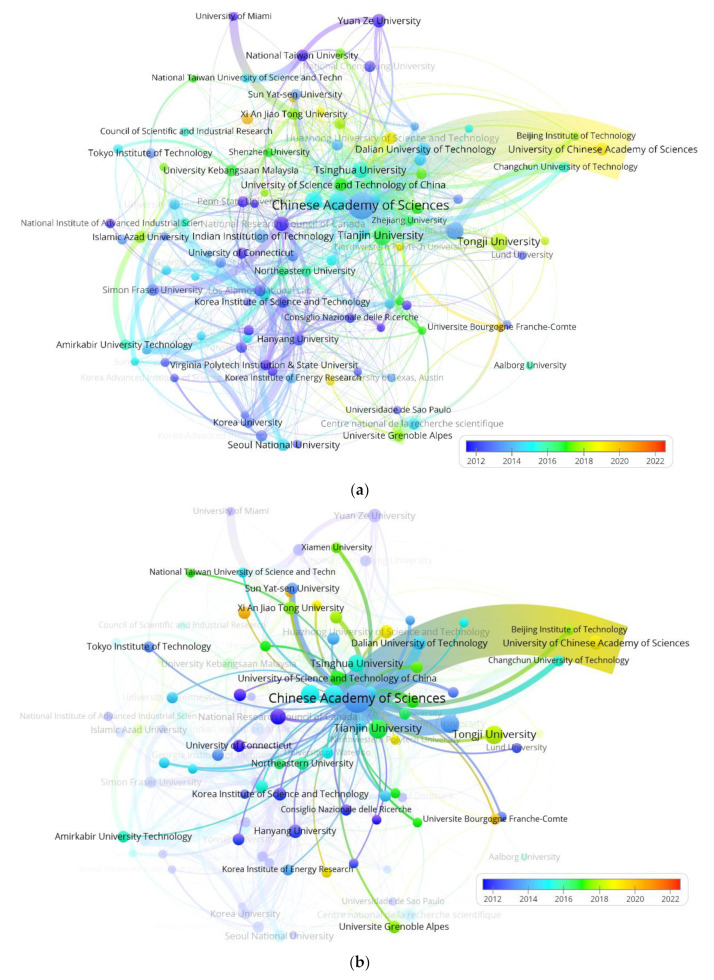

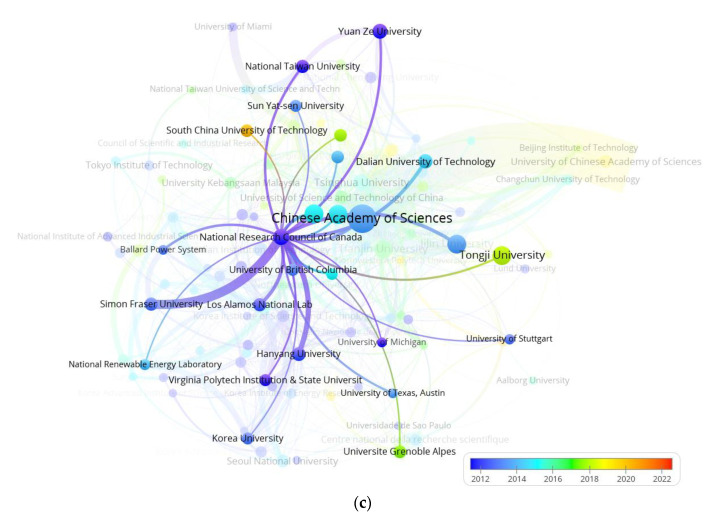
Map of the collaboration network of main institutions (**a**) the overall network (**b**) collaboration network with Chinese Academy of Sciences (**c**) collaboration network with National Research Council of Canada.

Furthermore, considering that the research field, comprehensive strength and publications of institutions make a difference in PEMFC research, it is necessary to gain insight into the contribution of institutions to PEMFC research from various countries. Therefore, it is necessary to carry out bibliometric study in terms of institutions. Figure 8a shows the overall collaboration network of main institutions. The size of circles and lines refer to publications and link strength, respectively, with colors indicating average publication year (APY). On the whole, institutions from China, the USA, South Korea and Canada cooperate closely. Before 2013, the National Research Council of Canada, Hanyang University in South Korea and Los Alamos National Lab in the USA published the most. After 2013, the Chinese Academy of Sciences, Tongji University, Shanghai Jiao Tong University and Tsinghua University are also prolific; their comprehensive strength have made them some of the top institutions in China. From the perspective of collaboration link strength, seen in Figure 8b, the Chinese Academy of Sciences, with the most publications, established the strongest collaboration with the University of Chinese Academy of Sciences domestically, and it cooperated more with Chinese and Korean institutions. As for overseas institutions from Figure 8c, the National Research Council of Canada cooperates most closely with Simon Fraser University, which is also in Canada.

Table 5 displays the summary information of top 10 institutions according to publication quantity. The top 5 institutions are all from China: Chinese Academy of Sciences with 765 publications, Tianjin University with 318 publications, Tongji University with 296 publications, Shanghai Jiao Tong University with 291 publications and Jilin University with 285 publications. Judging from this result, compared with research institutes in other countries, Chinese institutions pay more attention to PEMFC research and dominate in terms of publications. The contribution to PEMFC of Tianjin University is mainly made by the State Key Laboratory of Internal Combustion Engine Combustion.

### 3.4. Keywords Analysis

**Figure 9:**
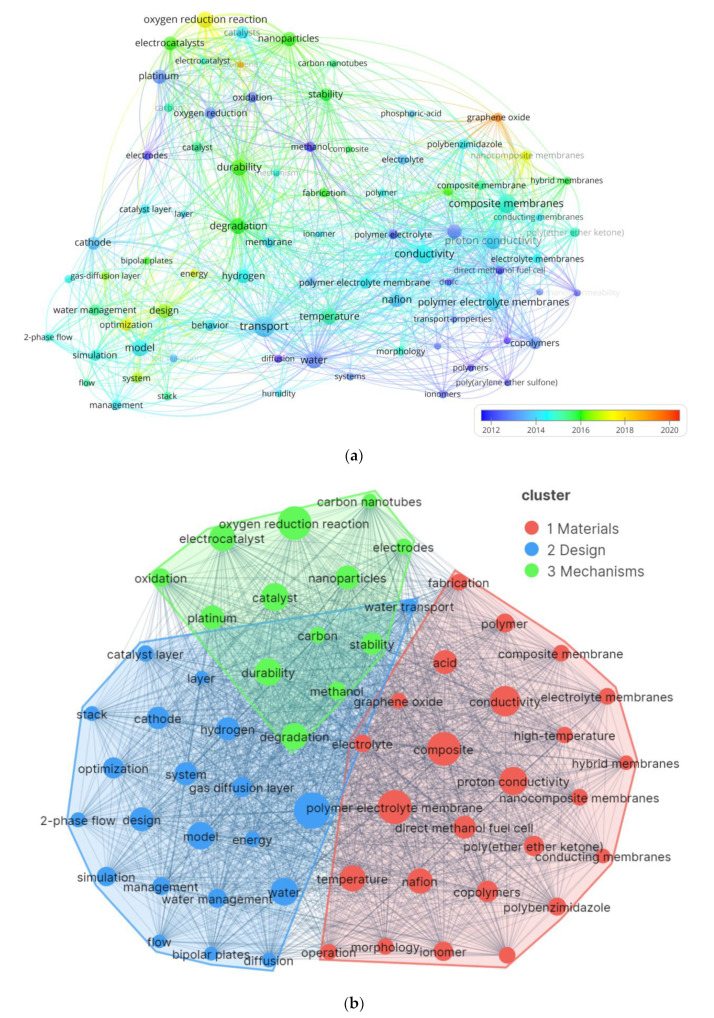
Keywords maps of PEMFC research (**a**) the overlay map of keywords (**b**) the keywords map in the distribution plot of three clusters.

By means of keywords analysis, the theme and trend of PEMFC research is easily acquired. When setting up parameters and choosing the “all keywords” option in the VOSviewer software, there are 58 keywords selected after setting the threshold for the frequency of keywords co-occurrence to 200. The results are presented in Figure 9a,b, which show the overlay map of keywords and the keywords map of PEMFC research in the distribution plot of three clusters, respectively. In Figure 9a, the color of circles refers to the average publication year of keywords. Some keywords like “graphene oxide”, “graphene” and “oxygen reduction reaction” have emerged in the recent years, which indicates new materials and mechanism development in the PEMFC field. According to bibliometric analysis, the three clusters can be divided into three aspects: “materials”, “design” and “mechanisms” in Figure 9b.

“Cluster 1 materials” mainly includes 26 high-frequency terms. Research on PEMFC can be summarized as preparation, modification and performance improvement of internal component materials, including new membrane materials and electrodes. The keywords of “polymer electrolyte membranes” [55], “nafion” [56], “composite membranes” [57] and so on, are related to the synthetic materials of PEMFC. Accordingly, the research on PEMFC materials has changed from traditional materials to new critical materials, while the keywords containing “conductivity” and “temperature” elaborate the characteristic of materials [58].

“Cluster 2 design” consists of 20 hot items, such as “transport”, “temperature” and “model”, involved in model simulation and management with respect to temperature and transport properties [59,60,61], which can be classified as design and application research for PEMFC. The structure design verification of PEMFC research relies on model simulation. Meanwhile, there are some difficulties that need to be take into consideration and solved from design to application. For instance, researchers have been attempting to provide the solutions of temperature control and transport management.

For “Cluster 3 mechanisms”, it is found from the research of “oxygen reduction reaction”, “catalyst” and “durability” as the keywords [62,63,64]. This is because the permeability of the membrane affects proton transport and battery durability, and the catalysts for oxygen reduction reaction under different operating conditions also affect the stability of the battery. Correspondingly, some researchers tried to outline the mechanisms concerning materials durability and the stability of proton transfer [65].

Furthermore, information of the top 10 keywords according to the occurrence ranking is demonstrated in Table 6. The most frequent keyword in the top 10 is “transport”, which belongs to the “cluster 2 design”, indicating that researchers have been challenging and solving the application design of PEMFC with respect to transport management. In addition, as presented in Table 6, keywords of the materials cluster are much more high-frequent, which also indicates the mainstream of PEMFC has always been materials research.

### 3.5. Limitations and Further Discussions

The bibliometric study in this article exists some limitations to some extent. On the one hand, from the perspective of data and methods, the data source is relatively singular, which is only obtained from the Web of Science without consideration of other databases, such as Scopus and Lens. Moreover, the maps generated by VOSviewer software only demonstrate a single style, and the algorithm is relatively weak when the data information is numerous. On the other hand, in terms of research findings, the number of publications by authors does not fully reflect their influence in the field of PEMFC research, and some authors are easily neglected in the map due to less links with others, which may result in omitting some representative literature by those authors on PEMFC. Moreover, the research in the field of PEMFC covers a wide range of topics, whereas the keywords based on bibliometric methods are relatively simple, which to some extent is not beneficial to making a comprehensive judgment on trends in the future.

Therefore, it is suggested that there is necessity to investigate more appropriate analysis methods and new software tools to conduct further bibliometric study of research on PEMFC. Moreover, owing to the technological development of energy and policy guidance, industry and academics in the field of PEMFC are closely linked; hence, increasing industry demand drives more academic achievements, encouraging the vigorous development of the industry, which may indicate that industry leads the development of academia in a good cycle. Therefore, the research field on PEMFC is gradually changing and becoming increasingly promising, and the information to be analyzed from the database is also constantly updating. In the future, bibliometric study should be more integrated and comprehensive for contributing to the progress of society.

## 4. Conclusions

In this study, bibliometric research on proton exchange membrane fuel cells was carried out. Data attained from the Web of Science Core Collection (WOS CC) database for publications between January 1990 and August 2022 were analyzed by the VOSviewer tool. From aspects of journal resource, authors, countries, institutions and keywords, the collaboration network, trends and geographical distribution were visualized and discussed in the results and discussion part.

In summary, PEMFC research has been developing rapidly worldwide. The representative authors are Shao and Yi, both of whom are prolific and have collaborated most closely with each other. In terms of the collaboration of countries and institutions, China and the USA have the strongest partnerships, and the University of Chinese Academy of Sciences cooperates most closely with the Chinese Academy of Sciences in China. Outside of China, the National Research Council of Canada cooperates most closely with Simon Fraser University. Additionally, the hot research direction in the PEMFC field is the types of PEMFC research that covers the whole process from preparation to application, which can be divided into three aspects: materials, design and mechanisms.

In conclusion, this study demonstrates that research on PEMFC is an increasing trend, and researchers worldwide have been pursuing solutions for improving the performance of the materials, strengthening application design and solving attenuation mechanism problems. In view of global energy development plans and urgent demands on PEMFC in various fields of application, the research of new materials and wider application design should be the development direction in the future.

## Figures and Tables

**Table 1:** Parameters used for the map in VOSviewer software.

Parameter	Value
minimum number of documents	non-negative value
minimum number of citations	non-negative value
minimum number of occurrences	non-negative value
attraction	an integer value between −9 and +10
repulsion	an integer value between −10 and +9
resolution	non-negative value
minimum cluster size	non-negative value

**Table 2:** Information of top 10 Journals in the PEMFC field.

Rank	Journal Source	Publications	APY	AC
1	International Journal of Hydrogen Energy	2244	2015.4	31.6
2	Journal of Power Sources	2078	2011.2	49.7
3	Journal of Membrane Science	770	2012.9	50.8
4	Journal of The Electrochemical Society	688	2011.3	63.2
5	Electrochimica Acta	508	2012.3	43.7
6	International Journal of Energy Research	303	2017.7	14.6
7	Fuel Cells	294	2013.5	24.5
8	Energy	268	2017.4	30.0
9	Applied Energy	257	2017.5	44.5
10	Energy Conversion and Management	245	2018.0	27.8

Note: APY = average publication year, AC = average citations.

**Table 3:** Publication information of authors in PEMFC field.

Rank	Authors	Countries	Publications	APY	TLS	TC	Research Fields
1	Shao, Zhigang	China	116	2015.1	277	3240	New materials
2	Yi, Baolian	China	113	2011.4	272	5155	Critical materials and attenuation mechanism
3	Jiao, Kui	China	112	2017.5	159	4300	Thermophysical problems
4	Pan, Mu	China	100	2014.1	128	3281	Electrode design and research
5	Na, Hui	China	94	2010.6	414	3715	Polymerized functional membrane
6	Zhao, Chengji	China	93	2011.9	367	3168	Polymer electrolyte membrane material
7	Tu, Zhengkai	China	82	2019.2	148	1560	Fuel cells and hydrogen production
8	Miyatake, Kenji	Japan	78	2013.3	116	5363	Proton-conducting membrane material

Note: APY = average publication year, TLS = total link strength, TC = total citation (counted as whole counts in the software version used in this study).

**Table 4:** Information on the top 10 prolific countries in the field of PEMFC.

Rank	Country	Continent	Publications	AC	APY	TLS
1	China	Asia	6518	27.8	2016.2	11,394,033
2	USA	North America	3043	65.8	2012.1	7,506,598
3	South Korea	Asia	1563	30.9	2013.9	4,526,906
4	Canada	North America	1015	57.6	2012.8	3,098,663
5	India	Asia	1003	25.9	2016.2	2,848,588
6	France	Europe	902	38.9	2014.5	2,580,333
7	Germany	Europe	715	59.3	2014.1	2,217,654
8	UK	Europe	509	41.7	2015.1	1,444,690
9	Japan	Asia	981	37.4	2012.1	2,959,912
10	Iran	Asia	686	24.8	2016.8	1,588,433

Note: AC = average citations, APY = average publication year, TLS = total link strength.

**Table 5:** Information summary of the top 10 institutions.

Rank	Institutions	Country	Publications	TC	APY
1	Chinese Academy of Sciences	China	765	28723	2013.8
2	Tianjin University	China	318	12028	2016.6
3	Tongji University	China	296	4095	2018.0
4	Shanghai Jiao Tong University	China	291	6571	2014.9
5	Jilin University	China	285	8388	2013.8
6	Wuhan University of Technology	China	282	7546	2015.2
7	Tsinghua University	China	252	8333	2015.6
8	National Research Council of Canada	Canada	191	20001	2009.6
9	Yuan Ze University	China	172	5094	2011.8
10	Indian Institute of Technology	India	170	5782	2013.9

Note: APY = average publication year, TC = total citations (counted as whole counts in the software version used in this study).

**Table 6:** Information summary of the top 10 keywords.

Rank	Keywords	Cluster	Occurrence
1	transport	design	2389
2	polymer electrolyte membrane	materials	1831
3	composite	materials	1803
4	oxygen reduction reaction	mechanisms	1794
5	conductivity	materials	1459
6	proton conductivity	materials	1273
7	catalyst	mechanisms	1220
8	water	design	1210
9	durability	mechanisms	1189
10	model	design	1188

## Data Availability

Not applicable.
